# Gene-flow investigation between garden and wild roses planted in close distance

**DOI:** 10.5511/plantbiotechnology.23.0708a

**Published:** 2023-12-25

**Authors:** Yuna Asagoshi, Eri Hitomi, Noriko Nakamura, Seiji Takeda

**Affiliations:** 1 Department of Agricultural and Life Science, Graduate School of Life and Environmental Sciences, Kyoto Prefectural University, Shimogamo Hangi-cho, Sakyo-ku, Kyoto 606-8522, Japan; 2 Research Institute, Suntory Global Innovation Center Ltd., Seikadai 8-1-1, Seika-cho, Kyoto 619-0284, Japan; 3 Biotechnology Research Department, Kyoto Prefectural Agriculture, Forestry and Fisheries Technology Center, Kitaina Yazuma Oji 74, Seika-cho, Kyoto 619-0244, Japan

**Keywords:** *APETALA2*, DNA marker, gene-flow, *KSN*, rose

## Abstract

Rose is a major ornamental plant, and a lot of cultivars with attractive morphology, color and scent have been generated by classical breeding. Recent progress of genetic modification produces a novel cultivar with attractive features. In both cases, a major problem is the gene-flow from cultivated or genetically modified (GM) plants to wild species, causing reduction of natural population. To investigate whether gene-flow occurs in wild species, molecular analysis with DNA markers with higher efficient technique is useful. Here we investigated the gene-flow from cultivated roses (*Rosa*×*hybrida*) to wild rose species planted in close distance in the field. The overlapping flowering periods and visiting insects suggest that pollens were transported by insects between wild and cultivated roses. We examined the germination ratio of seeds from wild species, and extracted DNA and checked with *KSN* and *APETALA2* (*AP2*) DNA markers to detect transposon insertions. Using two markers, we successfully detected the outcross between wild and cultivated roses. For higher efficiency, we established a bulking method, where DNA, leaves or embryos were pooled, enabling us to that check the outcross of many plants. Our results suggest that wild species and garden cultivars can cross in close distance, so that they should be planted in distance, and checked the outcross with multiple DNA markers.

## Introduction

Rose is one of the most important ornamental plants in the world. The global market size of roses is valued more than 10 billion USD, and the output in Japan was around 16 billion JPY per year, ranking the third after Chrysanthemums and Lilies in 2022 (MAFF, 2022). The genus Rosa contains 120 to 200 species, and historical breeding has generated hundreds of cultivars with variable flower morphology, color, scent, flowering time, and disease resistance. The whole genome of several wild species, *Rosa multiflora*, *R. chinensis*, and *R. rugosa*, has been sequenced, allowing molecular approaches for rose research and breeding (Nakamura et al. 2018; Raymond et al. 2018; Saint-Oyant et al. 2018; Zang et al. 2021).

Although classical breeding has produced a lot of attractive cultivars, this method is often limited to closely related plant species that can be crossbred. Recently, roses with novel character have been generated by transgenic method; for example, novel cultivar with blue color of petals is generated by expressing the flavonoid 3′,5′-hydroxylase gene derived from viola and is now available on markets (Katsumoto et al. 2007). With the completed genome sequences, it is expected that more attractive roses with novel feature are generated by genomic engineering including transgenic technology, cisgenesis, intragenesis, and genome editing (Kumar et al. 2020). While genetically engineered plants have various advantages, they can also have environmental risks. One of the major risks is gene flow to wild by pollen and hybridization with wild species, which had been reported not only in genetically modified (GM) plants but also in plants brought from outside (Ellstrand 2003; Kumar et al. 2020; Nakamura et al. 2011a).

For assessment of environmental risks, DNA-associated molecular markers have been used in several crops. For roses, a molecular marker associated with the *KSN* gene has been reported (Nakamura et al. 2011b). *KSN* gene is a homologue of *TERMINAL FLOWER 1*, and involved in repression of flowering out of winter to spring, so that wild species show once-flowering (OF) feature in spring. In a continuous-flowering (CF) cultivar *R. chinensis* Old Blush, a 9 kb transposon is inserted into the second intron of the *KSN* gene, suggesting that loss of function of the *KSN* results in release of repression so that this cultivar becomes perpetual flowering (Iwata et al. 2012). By examining the presence of the transposon insertion by PCR, it is possible to distinguish OF and CF cultivars; using this *KSN* gene marker, gene flow was investigated in cultivars and wild species, suggesting that no outcross from garden cultivars to wild species (mainly *R. rugosa*) in several distances (Nakamura et al. 2011b). Although this marker is useful to check whether roses carry the transposon in the *KSN* gene, it is a dominant marker so that it requires the positive control such as *GAPDH* gene, and it cannot distinguish heterozygous and homozygous genotypes for the transposon insertion.

Another DNA marker in roses has been reported using a gene associated with the double flower trait. Many cultivars have double flowers due to petalization of stamens and pistils, which is known to be caused by loss of function of the class C floral homeotic genes *AGAMOUS* (*AG*, Theißen and Saedler 2001; Yanofsky et al. 1990). Not only mutations within the *AG* gene itself, but also abnormalities in *AG* expression, such as DNA methylation in the *R.*×*hybrida* *AG* (*RhAG*) promoter region by low temperatures, cause suppression of *RhAG* expression, resulting in double flowers (Ma et al. 2015). Another example is the dominant mutant of *PETALOSA* (*PET*) gene, a member of the *APETALA2* (*AP2*)-like Target Of EAT-type (TOE) subfamily. The *PET* genes including *AP2* carry microRNA 172 (miR172) target sequence at their 3′-regions. Transposon insertion generates the transcripts lacking 3′-region including miR172 target site, so that the miR172 does not bind nor degrade *PET* gene, resulting in the PET dominant action to repress the class C function. According to this PET action, several plant species including roses show double flower phenotype (François et al. 2018; Gattolin et al. 2020, 2018). In roses, transposon is inserted to affect the miR172 binding, and the DNA marker that can be detected by PCR is successfully generated (François et al. 2018). This DNA marker enables us to detect both wild and double-flower genotypes in one reaction, so that is useful to analyze the outcross between wild single-flower species and double-flower cultivars.

To verify the gene diffusion of cultivars, we examined whether the cross occurred between wild species and garden cultivars planted in close distance. One of the problems was that it was time-consuming to extract DNA from each plant, so that we raised a high throughput method to examine the gene-flow to the wild species by bulking DNA, leaves, and embryos. We found that the PCR amplification was successful from embryo DNA, so that we can check the gene-flow without waiting for the seed germination.

## Materials and methods

### Plant materials, flowering period and flower-visiting insects

**Figure 1:**
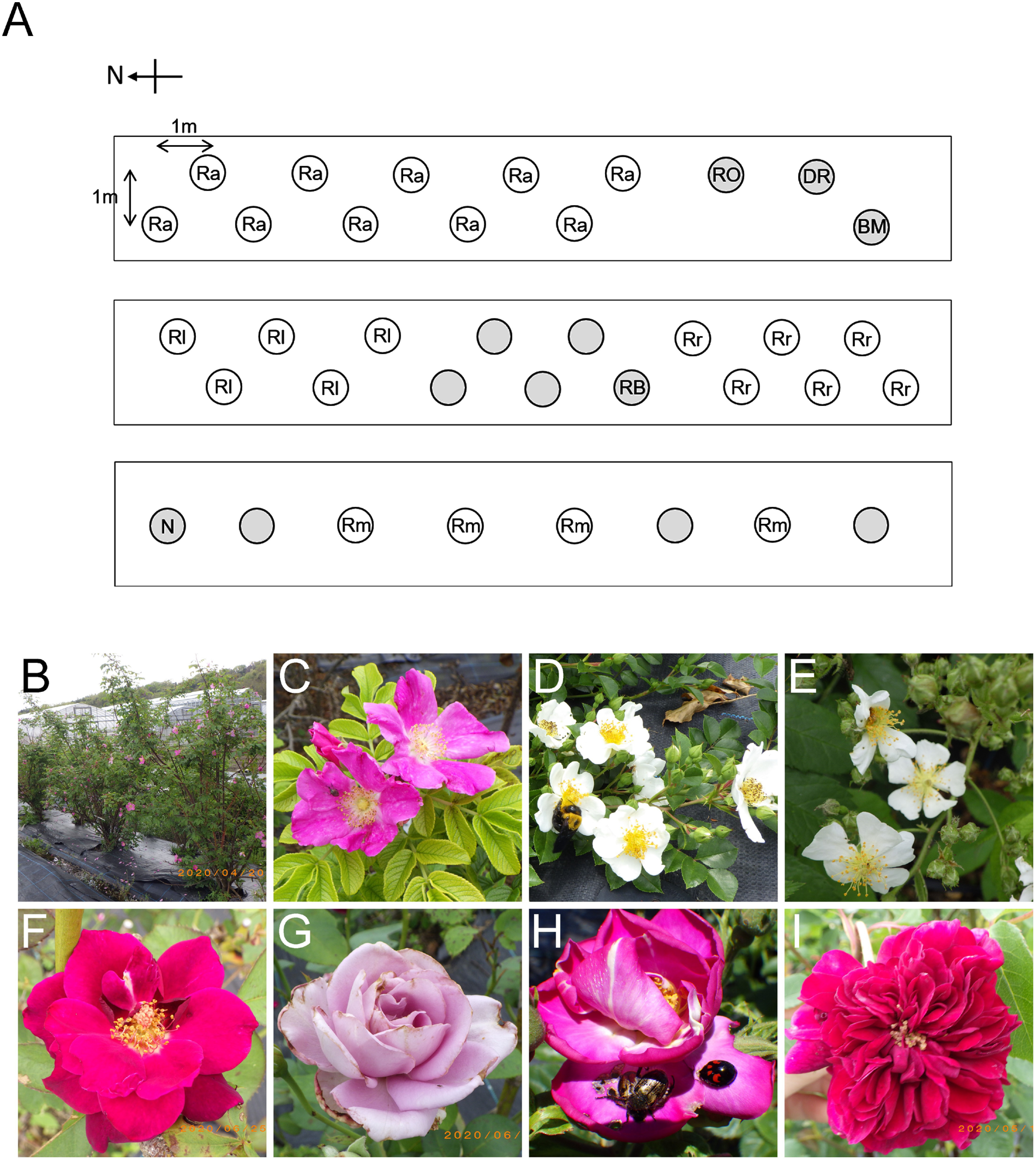
Figure 1. Field map and roses grown in this study. (A) Field map of roses closely planted. White and grey circles indicate wild species and garden cultivars, respectively. Ra: *Rosa acicularis*, Rl: *R. luciae*, Rm: *R. multiflora*, Rr: *R. rugosa*, RO: *Rosa*×*hybrida* cultivar ‘Royal’, DR: ‘Dramatic rain’, BM: ‘Blue moon’, RB: ‘Rhapsody in blue’, and N: ‘Novalis’. Empty circles indicate a breeding line. (B) *R. acicularis*. (C) *R. rugosa*. (D) *R. luciae*. (E) *R. multiflora*. (F) ‘Dramatic rain’. (G) ‘Blue moon’. (H) ‘Rhapsody in blue’. (I) ‘Royal’.

Four wild species (*R. multiflora* Thunb., *R. luciae* Rochebr. et Franch. Ex Crep, *R. rugosa* Thunb., and *R. acicularis* Lindl.) and 6 cultivated varieties of *R.*×*hybrida* (‘Royal’, ‘Blue moon’, ‘Dramatic rain’, ‘Rhapsody in blue’, ‘Novalis’, and a breeding line) were densely planted in the university agricultural field in Seika, Kyoto (Figure 1). Flowering periods and visiting insects were visually observed and photographed with digital and iPhone cameras.

### Seeds storage

Cynarrhodia (pseudofruits or rose hip) were collected from 2019 to 2022, and pulp was washed off with tap water to collect fruits. These fruits were wrapped in gauze, sealed in a zippered plastic bag with deionized water, and stored at 4°C. After 4 months, 10 fruits per pot were sown in containing Nippi Gardening Cultivation Soil (Nihon Hiryo), and placed in an ACP-7/SBR010T-18 chamber (Shimadzu Rika) at 25°C, with photoperiod of 16 h light and 8 h dark.

### DNA extraction and polymerase chain reaction

DNA was extracted from leaves (∼100 mg) of each cultivar using the DNeasy Plant Mini Kit (QIAGEN). KOD FX Neo (TOYOBO) and a MiniAmp thermal cycler (ABI) were used for the polymerase chain reaction (PCR). PCR reactions were followed as described before (François et al. 2018; Nakamura et al. 2011b). Primer sequences are listed in Supplementary Table S1. For DNA bulking, the extracted DNA from each cultivar or wild species was mixed in different ratio and used for PCR. For leaves bulking, leaf discs prepared with a cork borer (diameter 7.0 mm) were mixed and DNA was extracted as shown above. Embryos were removed from seeds and DNA from each embryo or bulked ones extracted using the DNeasy Plant Mini Kit (QIAGEN), the Edwards’ method (Edwards et al. 1991), or the one-step method (TOYOBO: https://lifescience.toyobo.co.jp/detail/detail.php?product_detail_id=164). There was no difference in the results among DNAs extracted by these methods.

## Results and discussion

### Flowering period and flower-visiting insects

Flowering period and flower-visiting insects of 4 wild species and 5 (2019–2021) or 6 (2022) garden cultivars were investigated from 2019 to 2022. Flowering started in April or May in all plants, and 3 wild species *R. acicularis*, *R. luciae*, and *R. multiflora* showed once-flowering feature, whereas *R. rugosa* and all cultivars showed continuous-flowering (Supplementary Table S2). A few flowers of *R. acicularis* bloomed out of the season in 2019 but not in the other years, probably due to the environmental effect in 2019, such as warm temperature in this season.

**Figure 2:**
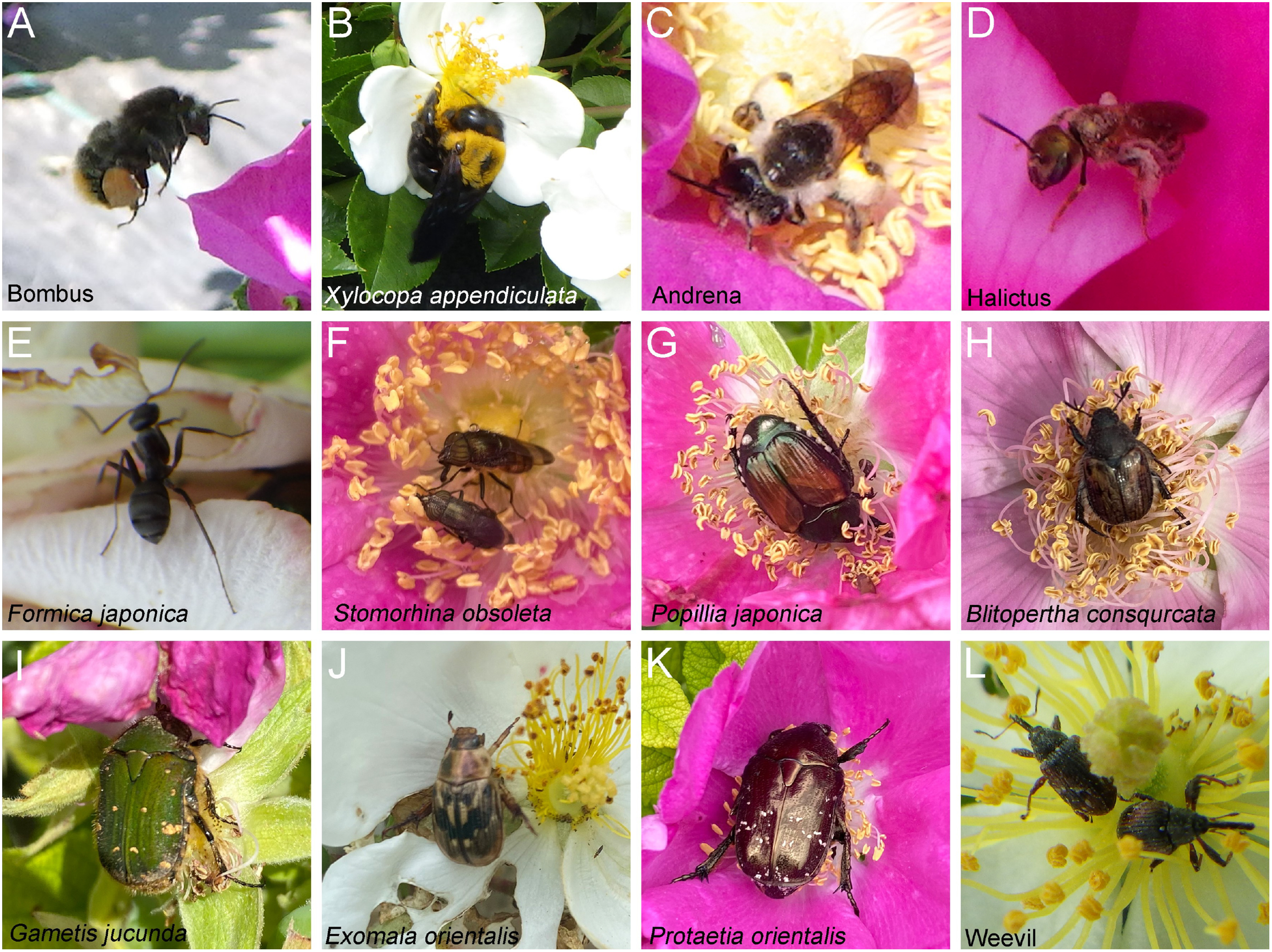
Figure 2. Flower-visiting insects. (A) Bumbus species. (B) *Xylocopa appendiculata*. (C) Andrena species. (D) Halictus species. (E) *Formica japonica*. (F) *Stomorhina obsoleta*. (G) *Popillia japonica*. (H) *Blitopertha consqurcata*. (I) *Gametis jucunda*. (J) *Exomala orientalis*. (K) *Protaetia orientalis*. (L) Weevil species.

We visually checked flower-visiting insects, and found that insects of the order Hymenoptera, Diptera, Coleoptera, and Hemiptera visited both cultivars and wild species (Figure 2). The overlapping flowering periods and visiting insects suggest that pollens were transported by insects between wild and cultivated species.

### Molecular markers to check the crossing between wild species and cultivars

**Figure 3:**
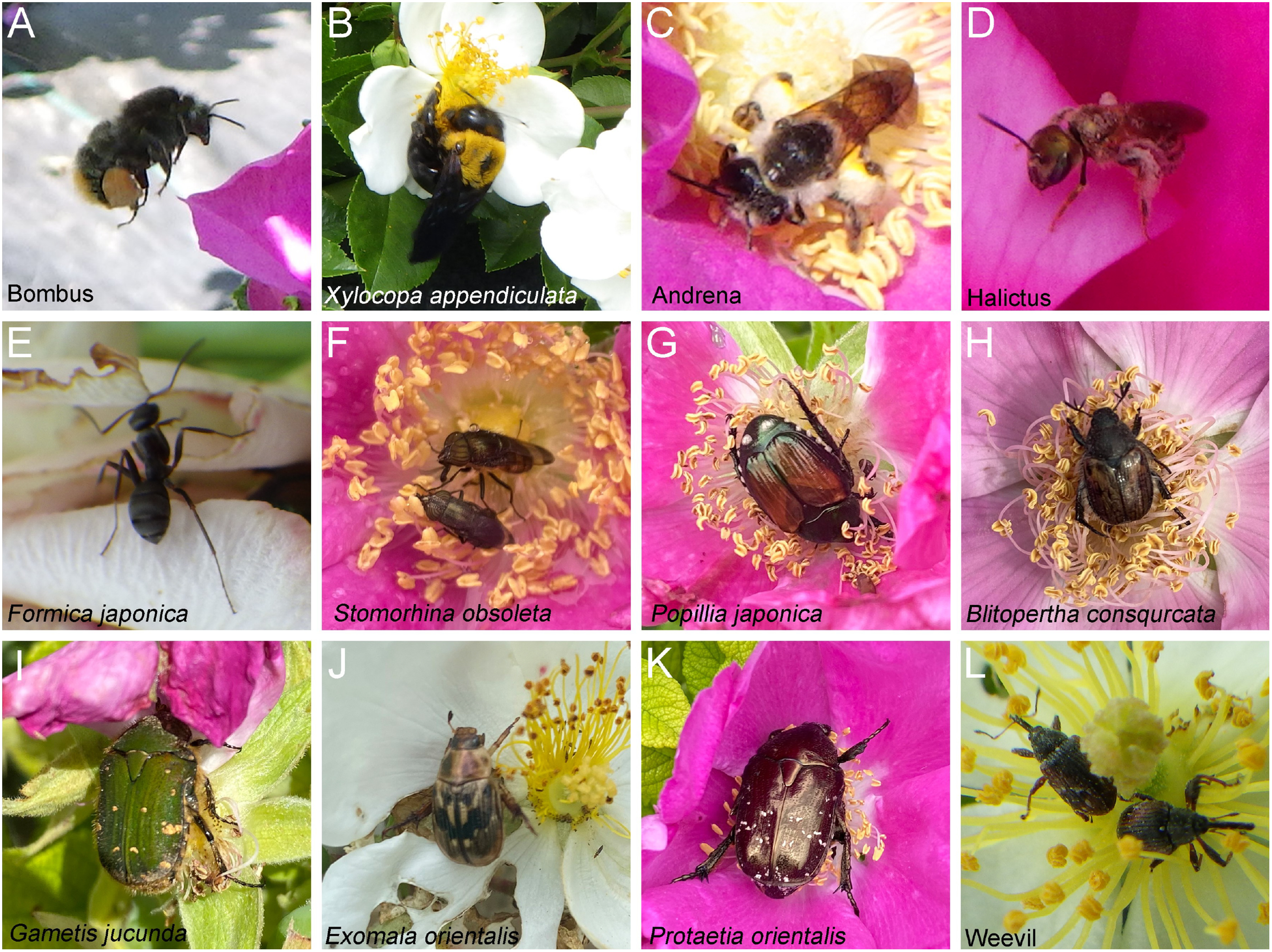
Figure 3. (A, B) PCR for amplification of *GAPDH* and *KSN* loci in wild species and garden cultivars. (C) PCR for amplification of *AP2* locus. Asterisks indicate non-specific amplicons. Rr: *R. rugosa*, Ra: *R. acicularis*, Rm: *R. multiflora*, Rl: *R. luciae*, RO: cultivar ‘Royal’, DR: ‘Dramatic rain’, RB: ‘Rhapsody in blue’, BM: ‘Blue moon’, Rh: *R.*×*hybrida* cultivar, N: ‘Novalis’, W: wild type, L: left border, R: right border.

To check the crossing between wild species and garden cultivars by molecular analysis, we first examined whether PCR-based DNA markers that had been reported worked for plants used in this study. Control PCR for *GAPDH* locus amplified the products of the expected band size from all wild species and cultivars, whereas *KSN* marker only did from garden cultivars (Figure 3A, B), consisting with the previous report that transposon was inserted in *KSN* locus in cultivars (Nakamura et al. 2011b).

We used another marker for the *AP2* locus, that amplifies the wild-type genotype (W, 419 bp), and left (L, 754 bp) and right (R, 770 bp) borders of the transposon insertion (François et al. 2018). We found that the *AP2* marker amplified the W band size product as expected in all plants, and L and R products in all cultivars (Figure 3C), suggesting that all cultivars are heterozygote for the *AP2* mutation (François et al. 2018). Unexpected L products with different molecular size were amplified in *R. luciae*, *R. multiflora*, and *R. acicularis* (Figure 3C, upper panel). We cloned the products of *R. luciae* and *R. acicularis* and sequenced, and found that it showed high homology to the ncRNA of *R. chinensis*, suggesting that they were non-specific products. To avoid confusion, we used W and R combination as an *AP2* marker hereafter.

### Bulking samples for efficient survey

**Figure 4:**
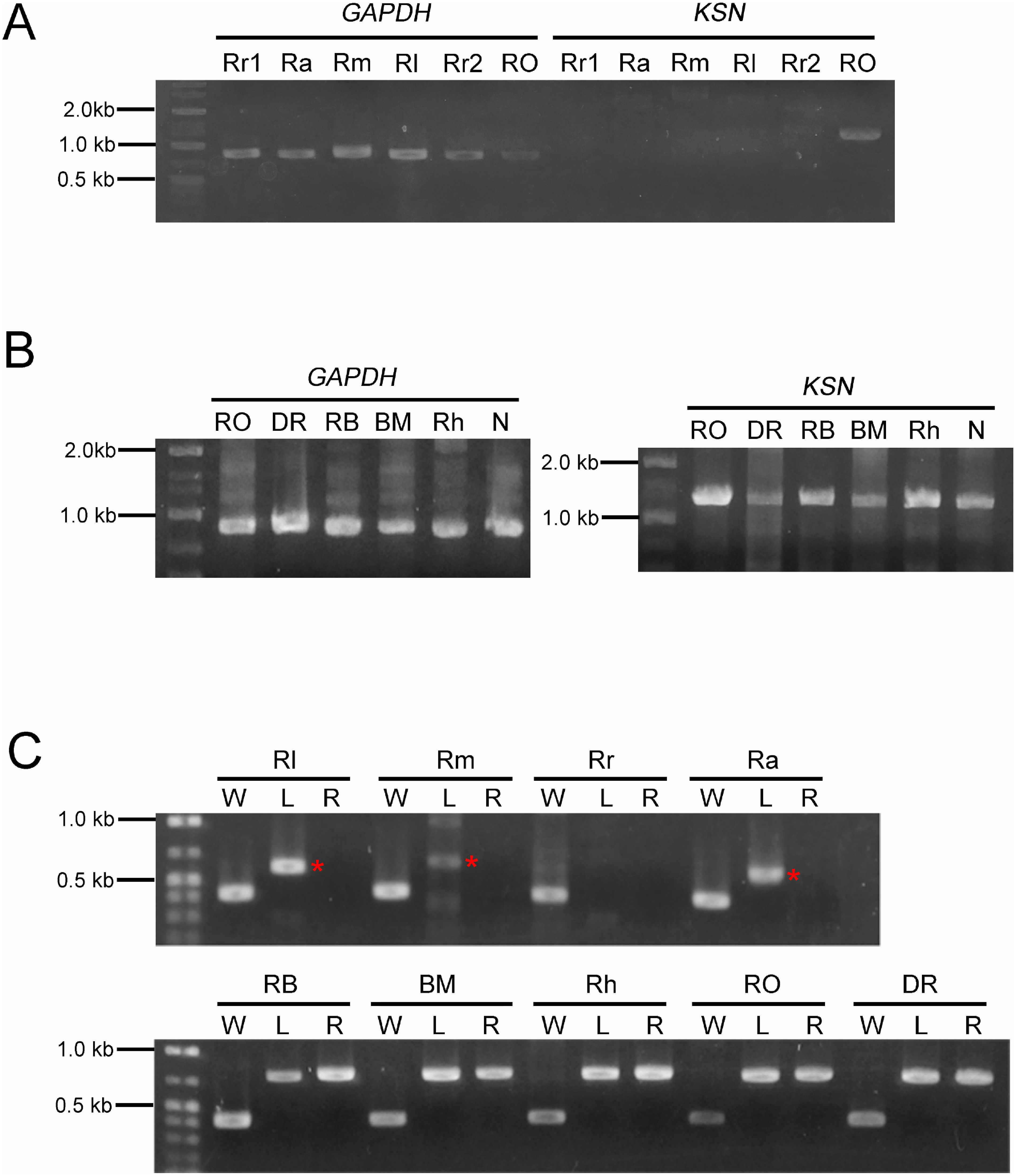
Figure 4. PCR with bulked samples, where DNA or leaves from wild species and garden cultivars were mixed in different ratio shown below the gel image. (A) DNA bulking. (B, C) Leaf bulking used for *KSN* and *GAPDH* (B) or *AP2* (C) markers. Rr: *R. rugosa*, Ra: R. acicularis, Rm: *R. multiflora*, Rl: *R. luciae*, RO: cultivar ‘Royal’, RB: ‘Rhapsody in blue’, W: wild type, R: right border.

To check the gene flow to wild populations, we need to check a lot of wild rose plants. For higher efficient survey, mixing the DNA or plant tissue from multiple plants is a good method to check many plant samples. We first examined whether the PCR worked with mixed DNA from wild species and garden cultivars. Bulked DNA sample with different ratio from wild species and garden cultivars were prepared, and analyzed by PCR, resulting in successful amplification of *KSN* locus when cultivar DNA ratio was as low as 2.5%, but no amplification from wild rose DNA (Figure 4A).

Although bulking DNA raises the efficiency for survey, it still requires individual DNA extraction from each plant. Therefore, we next examined whether PCR worked for DNA extracted from bulked leaves sample. We collected the leaf discs with a cork borer and mixed with different ratio for wild and cultivar roses, extracted DNA, and examined by PCR for the *KSN* and *AP2* markers, and found that DNA from the leaves bulking successfully amplified the expected products, even when the cultivar ratio was as low as 5% (*KSN*, Figure 4B) or 2.5% (*AP2*, Figure 4C). These results suggest that we can detect the gene-flow from the bulked leaves samples, making the gene-flow investigation with higher efficiency.

### PCR analysis with DNA extracted from embryos

In 2020, the pulp was removed from the harvested cynarrhodium and fruits including seeds were sown immediately, but the germination rate was extremely low (Table 1). Therefore, we wrapped the fruit with wet gauze after removing the pulp and stored at 4°C for 3 months or longer before sowing. The germination rate in 2021 to 2022 was higher than 2020, although it was still low (Table 1). The low germination ratio may be due to the duration of vernalization; it was too short in 2020, and too long in 2021 and 2022 (Gao et al. 2022). Although low germination ratio means less possibility of gene-flow to wild species, we cannot examine it without DNA from leaves.

**Table 1:** Table 1. Germination ratio of collected seeds from wild roses.

2020	
*R. multiflora*	1.77% (6/399)
*R. rugosa*	0.25% (1/394)
2021–2022	
*R. acicularis*	2.86% (2/70)
*R. luciae*	6.40% (16/250)
*R. multiflora*	16.19% (16/250)
*R. rugosa*	15.00% (2/70)

**Figure 5:**
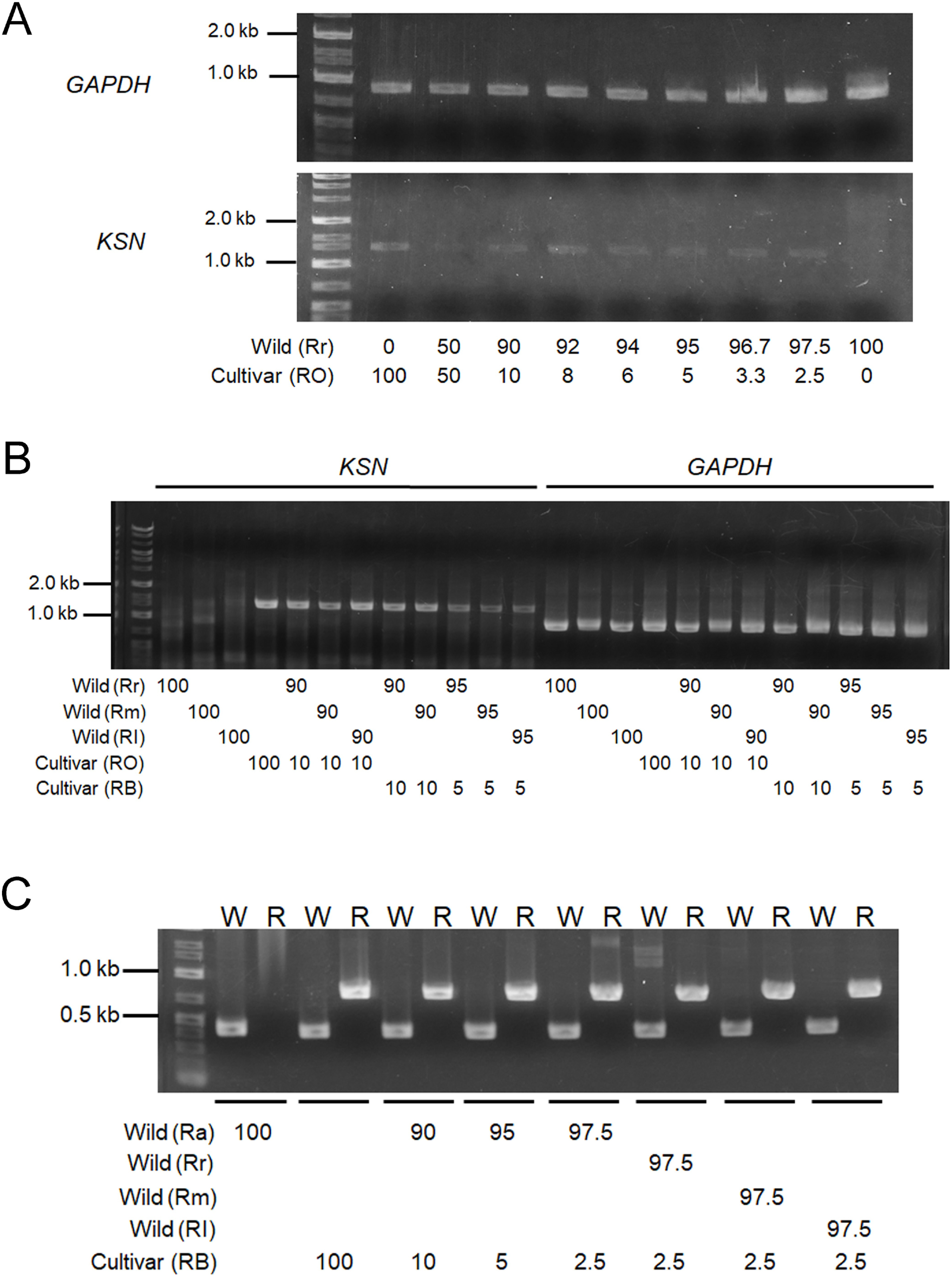
Figure 5. Embryo bulking. (A) White and plump embryo (arrowhead). (B) Black and small embryo (arrowhead). Scale bars, 1 mm. (C) PCR for *AP2* with DNA extracted from embryo of *R. acicularis* (Ra), *R.*×*hybrida* (Rh), and ‘Novaris’ (N). (D, E) Gene-flow investigation with bulked DNA. Note that *KSN* insertion was not detected in wild leaves (D), whereas AP2-R was detected in bulked embryo of *R. multiflora* and *R. rugosa* (E, red arrowheads). Rr, *R. rugosa*; Rm, *R. multiflora*; N, ‘Novalis’, W: wild type, R: right border.

Therefore, we took out embryos from seeds, extracted DNA and examined with the DNA markers. We found that there were two kinds of embryos; one was white and plump embryo, and the other was black and small (Figure 5A, B), and found that PCR amplified the expected bands from former embryos (Figure 5C), but not from latter ones. The latter embryos may stop developing and thus may not germinate, and this is one of the reasons for low germination ratio shown above.

### Gene-flow investigation with bulked DNA samples

Finally, we investigated the gene-flow from garden cultivars to wild species with bulked DNA from leaves germinated from seeds or embryos. From leaves DNA, we did not detect the PCR amplification either *KSN* or *AP2* markers (Table 2). We could not collect leaves from *R. acicularis* due to its extreme low germination ratio.

**Table 2:** Table 2. Gene-flow investigation with bulked DNA samples.

	Leaves		Embryos	
	Number of examined samples	Number of cross-detected samples	Number of examined samples	Number of cross-detected samples
*KSN*				
*R. acicularis*	0	0	37	0
*R. luciae*	0	0	0	0
*R. multiflora*	0	0	10	0
*R. rugosa*	0	0	87	0
*AP2*				
*R. acicularis*	0	0	31	0
*R. luciae*	10	0	10	0
*R. multiflora*	30	0	0	0
*R. rugosa*	20	0	26	0
*KSN* and *AP2*				
*R. acicularis*	0	0	27	0
*R. luciae*	30	0	2	0
*R. multiflora*	0	0	120	1–10*
*R. rugosa*	20	0	187	1–10*

*PCR amplification in 10 bulked samples.

Then we extracted DNA from bulked embryos and examined, and found the amplification of the garden cultivar-derived products (R band of AP2 marker) in *R. multiflora* and *R. rugosa* (Figure 5D, E, Table 2). We examined these samples with PCR for the *KSN* locus, but it did not amplify the products. This suggests that outcross occurred between wild roses and garden cultivars planted in close distance, the single parent was heterozygous for *KSN* locus (Bai et al. 2021; Horibe et al. 2015), and thus is better to check the gene-flow with multiple DNA markers.

## Conclusions

Since many cultivated roses have been generated from wild rose species, outcross between wild and cultivated roses can occur in nature. Nakamura et al. 2011b showed that outcross between wild and cultivated roses grown in several distances is likely to be very low by the *KSN* marker analysis. Our results suggest that using multiple DNA markers, *KSN* and *AP2*, raises accuracy for checking the outcross. Although we detected the outcross between wild and cultivated roses in very close distance, our results suggested that the cross between them is still very rare in wild, since (1) only a few crosses were detected in roses that were planted in very close distance, (2) generally they are not grown in such close distance, and (3) germination rate of wild rose seeds were very low. This means that we can expect few cases for outcrossing between wild and cultivated roses, still it is better to check the gene-flow to the wild, especially when we grow GM roses; DNA or leaves bulking allowed us to investigate more plants, and embryo bulking enabled us to investigate wild species with low germination ratio. In addition to them, the other DNA markers, e.g. that are linked to the disease-resistant locus (Biber et al. 2010; Zurn et al. 2020), and/or genes on transgene such as antibiotics-resistant gene, will be gain accuracy for gene-flow investigation. It is necessary to plant GM-crops away from wild species and isolate in greenhouse, and to check the gene-flow with DNA markers time to time, to prevent the gene-flow to wild populations.

## Abbreviations

*AP2*: *APETALA2*
CF: continuous-flowering
GM: genetically modified
OF: once-flowering
