# Supplementary material for: Gene-flow investigation between garden and wild roses planted in close distance
Source: Plant Biotechnol (Tokyo). 2023 Dec 25;40(4):283–8. doi: 10.5511/plantbiotechnology.23.0708a (PMC10905366; doi:10.5511/plantbiotechnology.23.0708a)

Supplementary Table S1. Primers used in this study

Genes	Primer name	Sequence (5'-3')	Reference
KSN	RhKSNIF3	CATATTATGGCATAGGGTGTGGC	Nakamura et al., 2011
	RhKSNInsR3	TGTAATCTGTAGGAGATCCCATGC	
GAPDH	RhGAPDH-237F	TGTCATCTCTGCCCCAAGTAAGG	
	RhGAPDH-724R	CAACATCCTCATCGGTGTAACCC	
AP2	RcAP2L_TE_right_border_F	GAAGCTGCAAGGTCTTTACGTTAAT	François et al., 2018
	RcAP2L_TE_right_border _R	GAGACTCTTCCACTTTGTTTCCTTG	
	RcAP2Lwt_F	ATTGCTATGTCATGTCTTCACAACC	
	RcAP2Lwt_R	TATGGATGGGAAAGAAGTTGGAGTT	

François L, Verdenaud M, Fu X, Ruleman D, Dubois A, Vandenbussche M, Bendahmane A, Raymond O, Just J, Bendahmane M (2018) A miR172 target-deficient AP2-like gene correlates with the double flower phenotype in roses. *Sci Rep* 8: 12912

Nakamura N, Tems U, Fukuchi-Mizutani M, Chandler S, Matsuda Y, Takeuchi S, Matsumoto S, Tanaka Y (2011) Molecular based evidence for a lack of gene-flow between *Rosa*×*hybrida* and wild *Rosa* species in Japan. *Plant Biotech* 28: 245–250

Supplementary Table S2. Flowering periods grown in the university field

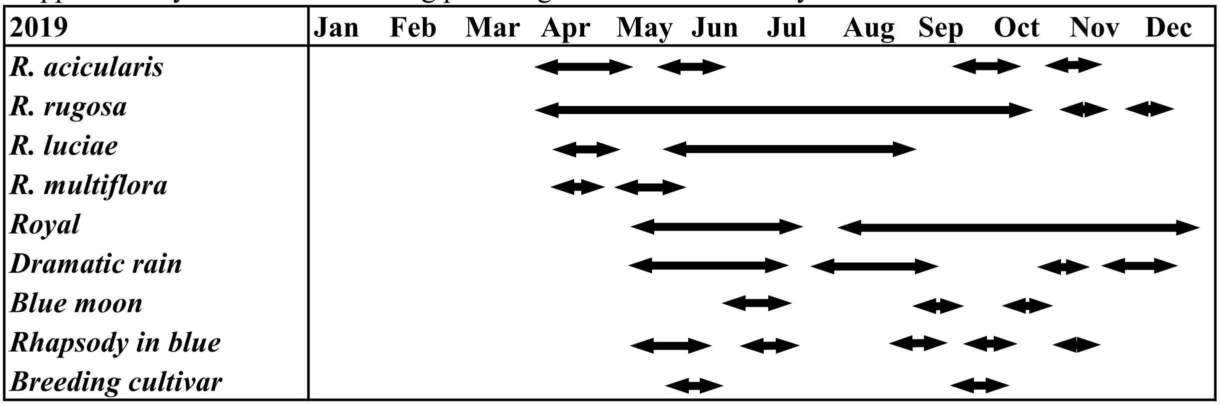

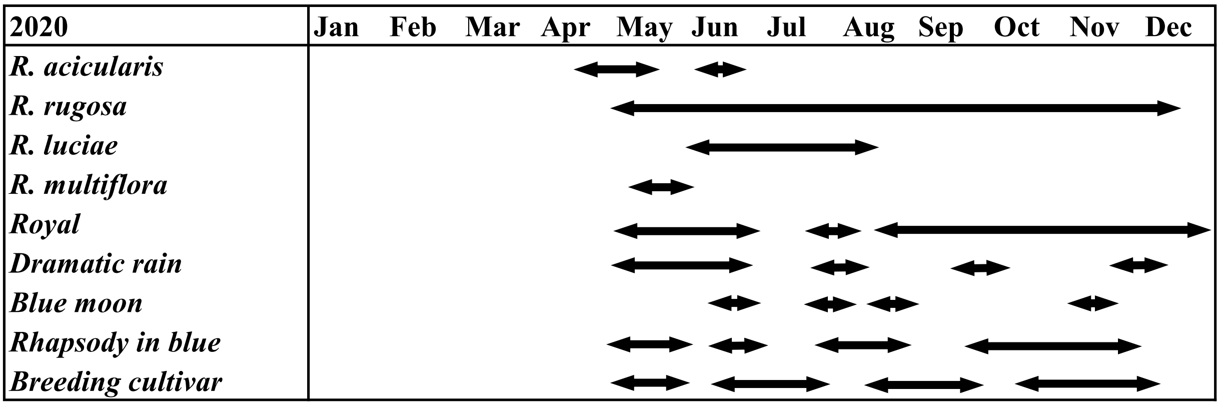

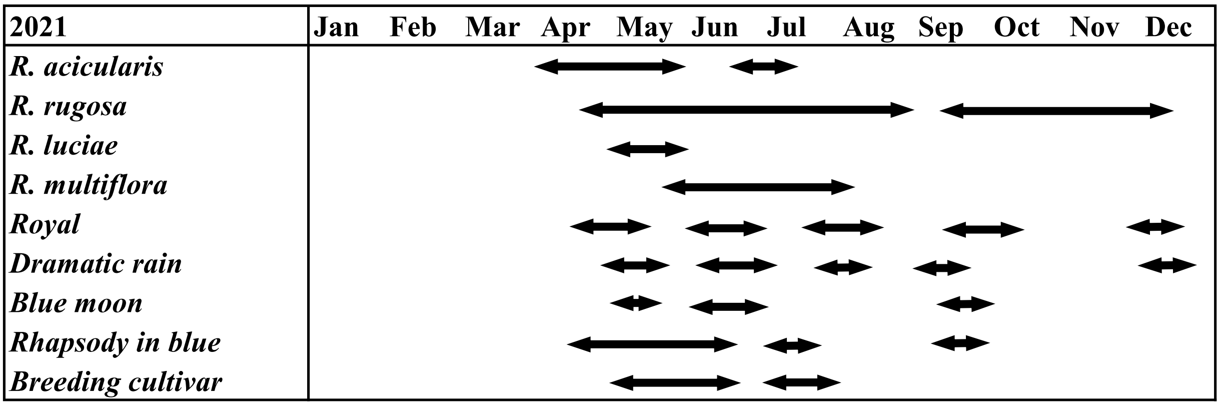

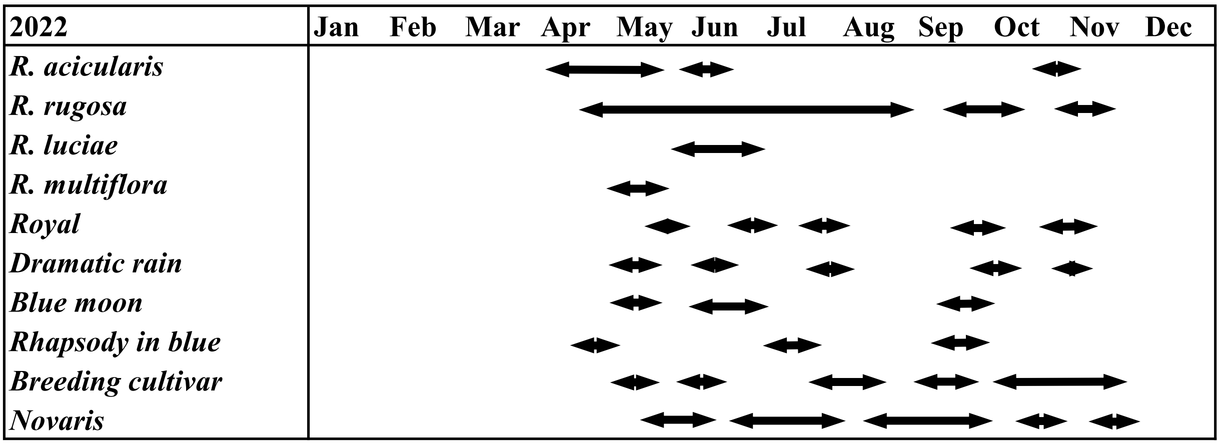

Supplement: Supplementary Data [file plantbiotechnology-40-4-23.0708a-s001.pdf]
